# Impact of severe systemic infections on neuroimmune interactions in neurodegenerative diseases

**DOI:** 10.1002/alz.090120

**Published:** 2025-01-03

**Authors:** Jonathan Anthony B. Villareal, Tim Bathe, Jennifer L Phillips, Stefan Prokop

**Affiliations:** ^1^ University of Florida, Gainesville, FL USA; ^2^ University of Florida / Center for Translational Research in Neurodegenerative Disease, Gainesville, FL USA; ^3^ University of Florida / Department of Pathology, Gainesville, FL USA; ^4^ University of Florida / Fixel Institute for Neurological Diseases, Gainesville, FL USA; ^5^ University of Florida / McKnight Brain Institute, Gainesville, FL USA

## Abstract

**Background:**

Severe systemic infections can trigger cognitive decline, but the underlying mechanisms and their impact on the manifestation and progression of Alzheimer’s disease and other neurodegenerative diseases are poorly understood. The current COVID‐19 pandemic has brought a surge of severe viral illness and highlights the importance of understanding the impact of acute infections on cognition and the manifestation of neurodegenerative disease in survivors. A wealth of observational and clinical data suggests major short‐ and long‐term effects of severe infections on cognition, but detailed and systematic analyses of neuropathological changes after acute infections are scarce. In order to bridge this gap and to provide insight into the cellular and molecular effects of severe systemic inflammation on hallmarks of neurodegenerative diseases, we performed immune profiling on the brains of recovered COVID‐19 patients and of patients without a history of severe systemic infection.

**Method:**

Immunohistochemical profiling of microglial, astrocytic, and oligodendrocytic morphological biomarkers was performed in human post‐mortem tissue sourced from various brain regions. Glial coverage was directly quantified using immunohistochemical analysis software QuPath with custom parameters designed to adapt the program for detecting the different cell types. Multiplexed gene expression analysis of immune and neuroglia‐related genes in human post‐mortem tissue was performed using the Nanostring nCounter platform.

**Result:**

Immunohistochemical profiling of glial cells in COVID‐19 patients revealed a subtle but distinct difference in microglial activation, as well as differences in coverage of other glial sub‐types, as compared to non‐COVID patients, while no significant differences in disease‐associated protein pathologies were observed. Multiplexed gene expression analysis corroborated these findings and also revealed significant shifts in oligodendrocyte and myelination‐related transcriptomic profiles in COVID patients. A spatial component of COVID‐19 was also observed, with a difference in severity of glial population shifts existing between brain regions.

**Conclusion:**

These findings demonstrate a subtle, but distinct and long‐lasting effect of severe systemic infections on the brain’s innate immune system which may impact the onset and progression of neurodegenerative diseases. Gene expression analyses of patients suffering from COVID‐19 also indicate significant alterations in the transcriptome of oligodendrocytes, and further investigation into the cross‐talk between these cells and microglia should be performed.

